# Malondialdehyde Suppresses Cerebral Function by Breaking Homeostasis between Excitation and Inhibition in Turtle *Trachemys scripta*

**DOI:** 10.1371/journal.pone.0015325

**Published:** 2010-12-22

**Authors:** Fangxu Li, Zhilai Yang, Yang Lu, Yan Wei, Jinhui Wang, Dazhong Yin, Rongqiao He

**Affiliations:** 1 State Key Laboratory of Brain and Cognitive Sciences, Institute of Biophysics, Chinese Academy of Sciences, Beijing, China; 2 College of Life Sciences, Hunan Normal University, Changsha, Hunan, China; 3 Laboratory of Mental Health, Institute of Psychology, Chinese Academy of Sciences, Beijing, China; 4 University of Science and Technology of China, Hefei, Anhui, China; New York State Institute for Basic Research, United States of America

## Abstract

The levels of malondialdehyde (MDA) are high in the brain during carbonyl stress, such as following daily activities and sleep deprivation. To examine our hypothesis that MDA is one of the major substances in the brain leading to fatigue, the influences of MDA on brain functions and neuronal encodings in red-eared turtle (Trachemys scripta) were studied. The intrathecal injections of MDA brought about sleep-like EEG and fatigue-like behaviors in a dose-dependent manner. These changes were found associated with the deterioration of encoding action potentials in cortical neurons. In addition, MDA increased the ratio of γ-aminobutyric acid to glutamate in turtle's brain, as well as the sensitivity of GABAergic neurons to inputs compared to excitatory neurons. Therefore, MDA, as a metabolic product in the brain, may weaken cerebral function during carbonyl stress through breaking the homeostasis between excitatory and inhibitory neurons.

## Introduction

Numerous studies have demonstrated that oxidation and glycation stresses are key causal factors of aging-related chronic diseases and neuronal degenerative diseases [1], [2], [3], [4], [5]. These stresses inevitably produce a variety of toxic intermediates, mainly unsaturated carbonyls, such as malondialdehyde (MDA) and formaldehyde (FA) [6], which usually cause carbonyl-amino crosslinking and eventually lead to the accumulation of irreversible pathophysiological changes (like lipofuscin) in the central nerve system (CNS) [7], [8], [9], [10]. The toxicity of such unsaturated carbonyls has been suggested as the important cause of aging-related biochemical alterations of animal and human body including brain degeneration [11], [12], [13], [14], [15]. Not only related with aging and diseases, the levels of toxic carbonyls have also been found largely increased following daily activities, particularly after intense exercise or sleep deprivation [8], [16].

Unsaturated carbonyls, like malondialdehyde (MDA) being less active than free radicals, often have a relatively longer half-life and, thereby, may diffuse from the places of generation to other sites in vivo, bringing further oxidative/carbonyl stresses. MDA, as one of the most important intermediates of free radical damages [8], [17], can readily and widely bind or crosslink important biological macromolecules such as proteins and nucleic acids [8], [18]. Such carbonyl stress-related reactions, or in simple, carbonylation, can form unstable 1∶1 amino-carbonyl compounds at an early stage and form versatile stabilized protein complex later [8], [10]. Although the early stage carbonylation can be actively reversed by several biological defense systems, the accumulation process of amino-carbonyl reaction continues, following daily activity or stresses [19], [20]. Therefore studies on the effects of carbonylation on the cerebral suppression and its relation to brain degeneration with respect of neuronal functions are of great interest and importance.

Although much work on carbonyl stress related to damage of animal brain has been reported, the effect of MDA on the turtle's cerebral activity is not reported. For instance, the LD_50_ of MDA for mouse is about 400 pmol/mouse (2 g/kg), suggesting that this compound is seriously toxic to mammals [21]. As we know, the turtle's brain appears to have stronger mechanisms to protect against the damages of reactive oxygen species than mice [22], [23]. Furthermore, turtle is able to resist to a relatively higher concentration of MDA administrated than mammals during the experiments [24]. This gives us a chance to observe the effect of a wide concentration range of MDA on the brain activities [25]. As far as it goes, the reports about the influence of carbonyls on the cerebral activities of the turtle have not been found, thus the turtle (*T. scripta elegans*) was employed in this investigation.

In this laboratory, as described previously, free radical damage to lipids resulted in malondialdehyde reacting with a protein and releasing an intermediate product of formaldehyde [26]. Formaldehyde at low concentrations induced protein misfolding to produce a high cytotoxic aggregation [6]. In this paper, we introduce turtle as a new model to study the effects of carbonyl stress induced by MDA on the CNS because this animal has the ability to be resistant to high levels of MDA.

## Methods

### Animals

Female 2-year-old red-eared turtles (*T. scripta elegans*) weighing between 900 and 1100 g (Changsha, China) were subject to the experiments. All animals were randomly assigned to eleven fiberglass tanks (0.8×0.4×0.5 m), which contained fresh water and a dry platform for thermal regulation. For acclimatization, they were housed in the experimental room for at least one week before study. Turtles were kept under the standard conditions with 12∶12 h light/dark cycles and at an ambient temperature of 23±1°C. Food was continuously available.

For EEG recordings, 20 turtles were randomly divided into 4 groups, whereas one group (5 turtles) was treated with control solution and other three groups (15 turtles) were treated with different concentrations of MDA. Thirty-six turtles were used for the assay of MDA concentration in turtles' brain. Thirty-six turtles were used for HPLC analyses to detect the changes of the GABA and Glu content in turtles' brain. Eighteen turtles were used for the whole-cell recordings. All experimental procedures were carried out according to regulations on the care and treatment of laboratory animals claimed by the Chinese Academy of Sciences. This study was approved by Bio-safety Committee and Animal Ethics Committee of Institute of Biophysics, Chinese Academy of Sciences (approval ID SYXK-125).

### Surgery

For intrathecal treatment of chemicals, a vertical hole (2.5 mm in diameter, 5–9 mm deep) at the center of backbone on the first median-back scale of the turtles was made with a sterilized tooth-drill. A small plastic catheter (1.5 mm in diameter, 10 cm long) connecting with a syringe (Kang-kang Medical Instrument Co. Ltd., Yuhuan, China) was inserted into the spinal cavity and fixed with dental cement. After the surgical procedures, the turtles were kept to recover for 3 days before the experiment.

### Preparation of MDA and control solution

MDA stock solution (200 mM, 4.05% w/w, the highest practical concentration to avoid possible self-polymerization of MDA under the experimental conditions) was freshly prepared by hydrolyzing 1,1,3,3-tetramethoxypropane (TMP) (purity≥98%, Fluka Chemie AG, Buchs, Switzerland), which was modified according to the method as described [19], [27], [28]. Namely, 338 µl TMP was mixed with 1.5 ml 1 M HCl, and shaken at 40°C to obtain MDA through acidic hydrolysis. After the solution became homogeneous, indicating a successful hydrolization of TMP, the acidity of solution was carefully neutralized to pH 7.2 with 6 M NaOH and made up to 10 ml with ultra pure water. Diluted with 0.10 M phosphate buffer, MDA working solutions (final concentrations, 0.2 µM, 2 µM and 20 µM) were injected (100 µl/kilogram) into spinal cavity injection. The control solution was prepared by substituting TMP with the saline solution (1 M HCl, 6 M NaOH, pH 7.2, 10 ml).

### EEG recordings and behavioral observations

Quantified EEG analysis is a sensitive and reproducible technique in assessing the effects of drugs acting on CNS during sleep-wake cycles [29], [30], [31]. The EEG signals were measured with KT88-AV2000 BEAM System (Contec Medical Systems Co. Ltd., Alhambra, USA). Stainless steel needles (0.1 mm in diameter, Suzhou Medical Appliance Factory, Suzhou, China) were used as electrodes to detect the EEG signals. One electrode was inserted into the nasion as a reference electrode and other two electrodes were symmetrically inserted into the right and left occipital regions of the skull (3 mm lateral and 3 mm anterior to the lambda).

The EEG of turtles was recorded for 30 and 40 minutes separately before and after MDA treatment. The speed of MDA infusion was maintained at 0.10 ml/kg/5 minutes. During EEG recordings, the turtles were fixed on an experimental platform with cellulose tapes and isolated in an observable cabin with ventilation system. The room temperature was maintained at about 23±1°C.

EEG signals were amplified and converted into digital signals by means of a multi-channel A-D converter and processed for fast Fourier transform spectral analysis. The sampling rate for EEG was 128 Hz and the signals were bandpass-filtered between 1.0 and 30.0 Hz before analysis. EEG amplitude was 50 µV/cm. The EEG power density in four frequency bands, delta (1.0–4.0 Hz), theta (4.0–8.0 Hz), alpha (8.0–13.0 Hz) and beta (13.5–30.0 Hz) waves, was calculated from data of 5 turtles, whereas spectral data of 3 individual epochs (30 s for each epoch) were obtained from each turtle (epochs containing artifacts were omitted).

Heart rate was recorded from inserting an additional electrode into left posterior leg of the turtle. The behavior of the whole animals was closely observed. To judge the vigilance states of turtles, the threshold of turtle's reaction to stimuli was checked by sticking the left posterior leg of the turtle with a needle (the needle-stick test). The needle-stick test was performed at every 10 minutes after MDA treatment.

### The assay of MDA concentration in turtles' brain

The turtle brain was homogenated by a homogenizer with 9 folds 0.86% NaCl on a glacial board. The cerebral homogenates were centrifuged (2000 rpm, 4°C, 15 minutes). The supernatant fluid (the cerebral extract) was used for the assay of MDA concentration. The method was as described by Kikugawa and coworkers [32].

### Whole-cell recordings

Cerebral slices (400 µm) were prepared from red-eared turtles that were decapitated quickly with a guillotine. Cerebral slices were cut with Vibratome in the oxygenated (95% O_2_ and 5% CO_2_) artificial cerebrospinal fluid (ACSF, mM: 100 NaCl, 3.5 KCl, 1.25 NaH_2_PO_4_, 26 NaHCO_3_, 2 CaCl_2_, 2 MgSO_4_, and 2 glucose, pH 7.4) at 40°C. The slices were held in ACSF at 25°C for 1–2 hours. A slice was transferred to a submersion chamber (Warner RC-26G) that was perfused with the oxygenated ACSF at 31°C for whole-cell recording as described [33]. Chemicals were from Sigma. The procedures were approved by IACUC in Beijing, China.

Pyramidal neurons show pyramidal-like soma and an apical dendrite, and interneurons are round with multipolar processes under DIC optics (Nikon FN-E600, Japan). Pyramidal neurons and interneurons show the different properties in the response to the hyperpolarization and depolarization pulses, especially spike patterns.

The recordings were conducted in current-clamp model with an Axoclamp-2B amplifier (Axon Instrument Inc., Foster CA, USA), and electrical signals were inputted into pClamp 10 (Axon Instrument, Inc, Foster CA, USA) for data acquisition and analysis. Output bandwidth in amplifiers was 3 kHz. The spike patterns were evoked by depolarization current pulses, in which the amplitude and duration were based on the aim of experiments. Pipettes for whole-cell recordings were filled with the standard solution that contained (mM) 150 K-gluconate, 5 NaCl, 5 HEPES, 0.4 EGTA, 4 Mg-ATP, 0.5 Tris-GTP and 5 phosphocreatine (pH 7.35 adjusted by 2 M KOH). Fresh pipette solution was filtered with centrifuge filters (0.1 µm pores) before use, and the osmolarity was 295-305 mOsmol. Pipette resistance was 5–6 MΩ.

Inter-spike intervals (ISI) are used to define spike capacity at threshold stimulus. A small ISI indicates that the cells can fire more spikes in a given time. ISI or the standard deviation of spike timing (SDST) of sequential spikes is linearly correlated with absolute refractory period (ARP) or threshold potential (ΔVts = Vts-Vr), indicating that ARP and ΔVts control the capacity of sequential spikes. We examine whether MDA affects ISI and SDST of sequential spikes at pyramidal neurons and interneurons through changing ARP and ΔVts.

The intrinsic properties of cerebral pyramidal neurons and interneurons in our investigation include the threshold potentials for initiating spikes and refractory periods following each of spikes. The threshold potentials are a start point of the rising phase of spikes [33]. The ARP of sequential spikes are measured by injecting multiple depolarization current pulses (3 ms and 5% above threshold levels) into the neurons following each of spikes. By changing inter-pulse intervals, we define ARP as duration from a complete spike to a subsequent spike at 50% probability [33]. Spike capacity is represented as ISI.

### HPLC of cerebral neurotransmitters in turtles' brain

The standard solution of GABA or Glu (1 mM) was mixed with water-methanol (1∶1) and stored at 4°C. The standard stock solutions were freshly diluted with Ringer solution as described previously [34].

The turtles were decapitated at different time intervals (5 and 35 minutes) after MDA or control treatment. Their brains were quickly taken out, and homogenated by a homogenizer with methanol on a glacial table. The cerebral homogenates were sonicated, and then centrifuged (12000 rpm, 4°C, 20 minutes). The supernatant fluid (the cerebral extract) was used for HPLC. *o*-Phthalaldehyde (OPA, 25 mg) was dissolved in 0.4 M borate buffer (5 ml, pH 9.6) containing 0.5 ml methanol anhydrous and 40 µl β-mercaptoethanol before use. The derivatization was carried out by adding the OPA solution (30 µl) to the standard amino acid solution or the cerebral extract of turtle brain at equivalent volume (reaction time, 1.5 minutes). After that, 50 µl of the reacted sample was taken for HPLC.

The sample was injected into a Venusil MP ODS column (250×4.6 mm I.D., 5 µm particle size, Agela Technolodies Inc, Newark, USA) to run the gradient elution by a pump (Hitachi 7100, Japan) and monitored (338 nm, 37°C) by an ultraviolet detector (Hitachi 7420, Japan). At pH 7.2 (adjusted with acetic acid), mobile phase A (1000 ml) containing 2.72 g sodium acetate anhydrous/0.2 ml triethylamine and mobile phase B containing 1.35% (w/w) sodium acetate anhydrous-acetonitrile-methanol (4∶7∶9, v/v) were filtered through a 0.22 µm polyamide membrane filter and degassed with the ultrasonication (SB-5200 ultrasonic cleaner, Xin-zhi Biological Instrument Co. Ltd., Ningbo, China) for 10 minutes. The derivative product was separated with the gradient elution with the mixture of the mobile phase A+B as indicated with the volume proportion of the mobile phase B: 4 minutes from 0.5% to 26%, 4 minutes at 26%, 7 minutes from 26% to 100%, and finally another 7 minutes at 100%.

### Statistical analysis

Data of EEG recordings and HPLC analysis were analyzed and showed in means ± standard deviation (S.D.). Student t-test was used to compare mean values of EEG power density, HPLC peak area of Glu and GABA and the ratio of them, and the MDA concentration in order to assess the statistical significance of the changes. Data of whole-cell recordings were analyzed when the recorded neurons had the resting membrane potentials negatively more than −55 mV. The criteria for the acceptation of each experiment also included less than 5% changes in resting membrane potential, spike magnitude, input resistance and seal resistance throughout each of experiments. Input resistance was monitored by measuring cell responses to the hyperpolarization pulses at the same values as the depolarization that evoked spikes. ΔVts, ARP and ISI are presented as mean ± SE. The comparisons between groups are done by paired t-test. The statistical analysis was performed using the software package Origin, version 8.0 for Windows.

## Results

### EEG recordings and behavior changes

**Figure 1:**
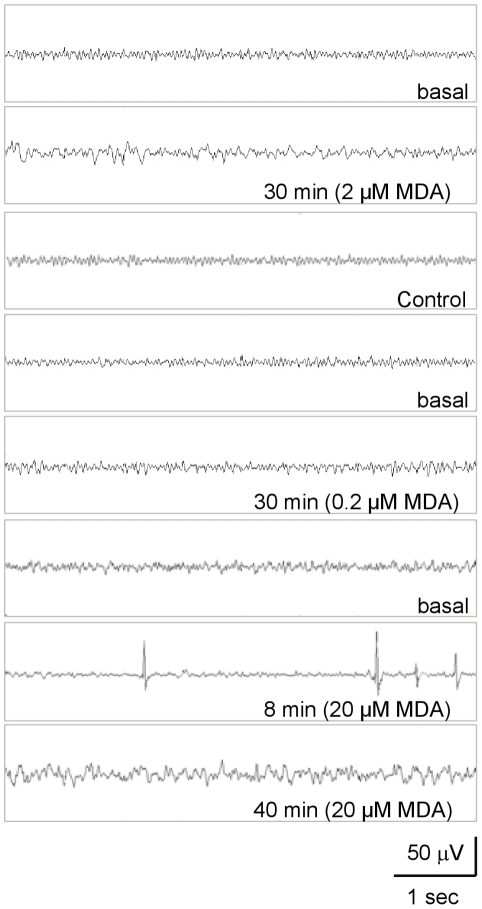
Effects of different concentrations of MDA on the EEG signals of turtles. Typical EEG spectra of turtles treated with different concentrations of MDA (0.1 ml/kg body weight) at different time intervals were as indicated. The EEG signals of turtles without MDA treatment were used as control.

Before MDA treatment, the turtles' heads lifted, and their eyes opened. They occasionally moved their heads and legs, with intense muscular activities. The threshold for the reaction to stimuli was low, showing a quick retraction of heads and legs into the shell. Under this condition, the EEG was characterized by a pattern of high frequency and low amplitude (control in Fig. 1).

Malondialdehyde (final concentration, 2 µM/kg body weight) was intrathecally injected into the spinal cavity of the turtle, and slow waves with high amplitude appeared after a latency of 5–8 minutes (Fig. 1). This state maintained for about 1 h and then the EEG approached to the basal state. Note that the heart rates (24 beats/min on average) of the turtles did not show statistically significant changes. Under 2 µM MDA treatment, the eyelids and heads of turtles were not observed to hang down distinguishably. Their reaction threshold remained relatively low and the animals responded (retracting heads and legs) quickly to stimuli. To confirm the effect of 2 µM MDA treatment on turtles, MDA treatment (0.2 µM/kg) was carried out. Neither the EEG signals nor the heart rates including the behavior of turtles showed significant changes compared to control group.

**Figure 2:**
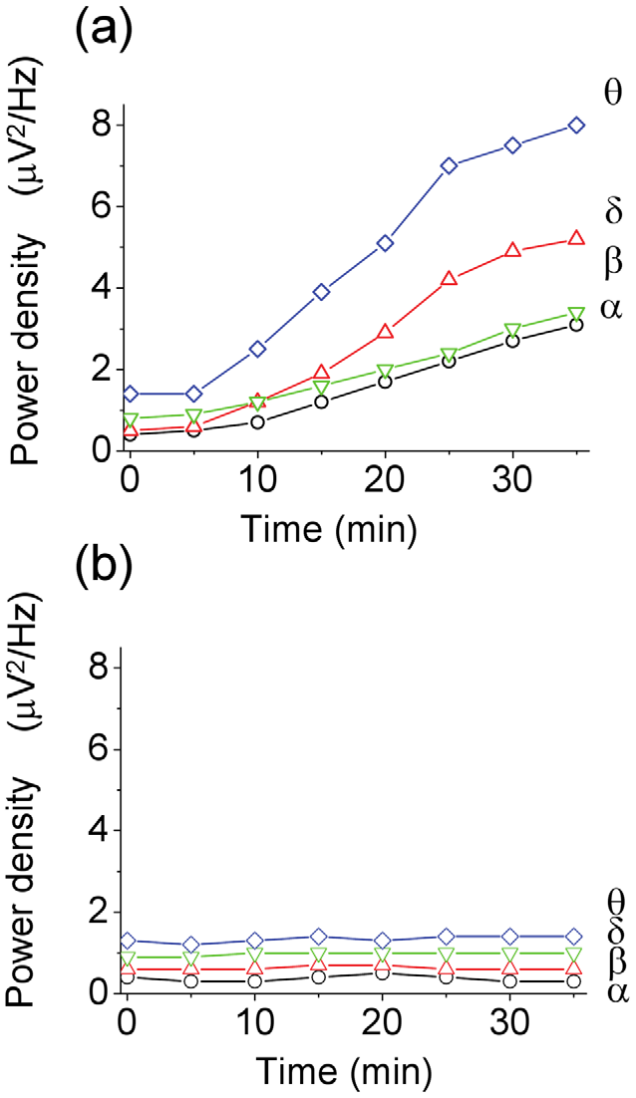
Quantified EEG power density analysis before and under the MDA treatment. The time course of EEG power density of different frequency was monitored under 20 µM MDA treatment (panel a) and that of control (panel b). Data were means from 15 turtles (n = 15), respectively. The statistic data are shown in Tab. 1.

In order to see a strong effect of MDA on the behavior and EEG, a high MDA concentration treatment was performed. Similar but stronger EEG changes were observed when turtles were treated with 20 µM/kg MDA than that with 2 µM MDA (Fig. 1). The intrathecal treatment of 20 µM/kg MDA induced, with a latency of 3–5 minutes, an almost complete suppression of EEG signal with irregular spike waves. This period lasted for 2–3 minutes on average. Subsequent EEG tracings were characterized by slow waves that gradually increased in amplitude to reach values remarkably higher than basal waves as control. Quantified EEG analysis revealed statistically significant changes of power density in all 4 frequency bands (Tab. 1 and Fig. 2). The heart rates after 20 µM MDA treatment (16 beats/minutes on average, also showed a significant change, compared with those before the treatment (24 beats/minutes on average). Under 20 µM MDA treatment, some turtles rested their heads on the experimental platform, with relaxed necks, closed eyes, and extended legs out of their shells. Their reaction threshold to stimuli was remarkably increased after the treatment. These results indicate that the carbonyl stress significantly affect the cerebral activity of the turtles in a concentration dependent manner, suggesting a suppression of the animal related to MDA.

**Table 1:** Statistical significance of the changes in power density after 20 µM/Kg MDA treatment versus basal condition. (n = 15).

Time(min)	Delta band	Theta band	Alpha band	Beta band
5	n.s.	n.s.	n.s.	n.s.
10	P<0.001	P<0.001	P<0.05	P<0.05
15	P<0.001	P<0.001	P<0.001	P<0.001
20	P<0.001	P<0.001	P<0.001	P<0.001
25	P<0.001	P<0.001	P<0.001	P<0.001
30	P<0.001	P<0.001	P<0.001	P<0.001
35	P<0.001	P<0.001	P<0.001	P<0.001

### MDA concentration in turtles' brain after administration

To confirm the suppression in the presence of MDA, changes of the MDA concentration in the turtle's brain was detected. Changes in the MDA concentrations in the turtles' brain (5 minutes and 35 minutes) were observed (the EEG had also significant changes as mentioned above). As shown in Tab. 2 **,** the MDA end-concentrations of 35 minutes group after 2 µM or 20 µM treatment are statistically higher than those of control group and 0.2 µM group (p<0.001). This is to say, the MDA diffused into the turtle's brain from the spinal cavity and the MDA level became higher than the normal level after 5 minutes of the injection. The MDA level was markedly increased around 35 minutes.

**Table 2:** MDA concentration in turtle brain at 5 min and 35 min after MDA or control treatment. (Means±SD, n = 3) (Unit =  μM/Kg).

Time(min)	20 µM MDA	2 µM MDA	0.2 µM MDA	control
5	3.54±0.83	3.36±0.87	2.97±0.78	3.57±0.84
35	48.09±6.64*	37.79±6.35*	3.37±0.92	3.34±0.77

*p<0.001 vs control.

### MDA deteriorates neuronal encodings in the brain of the red-eared turtles

**Figure 3:**
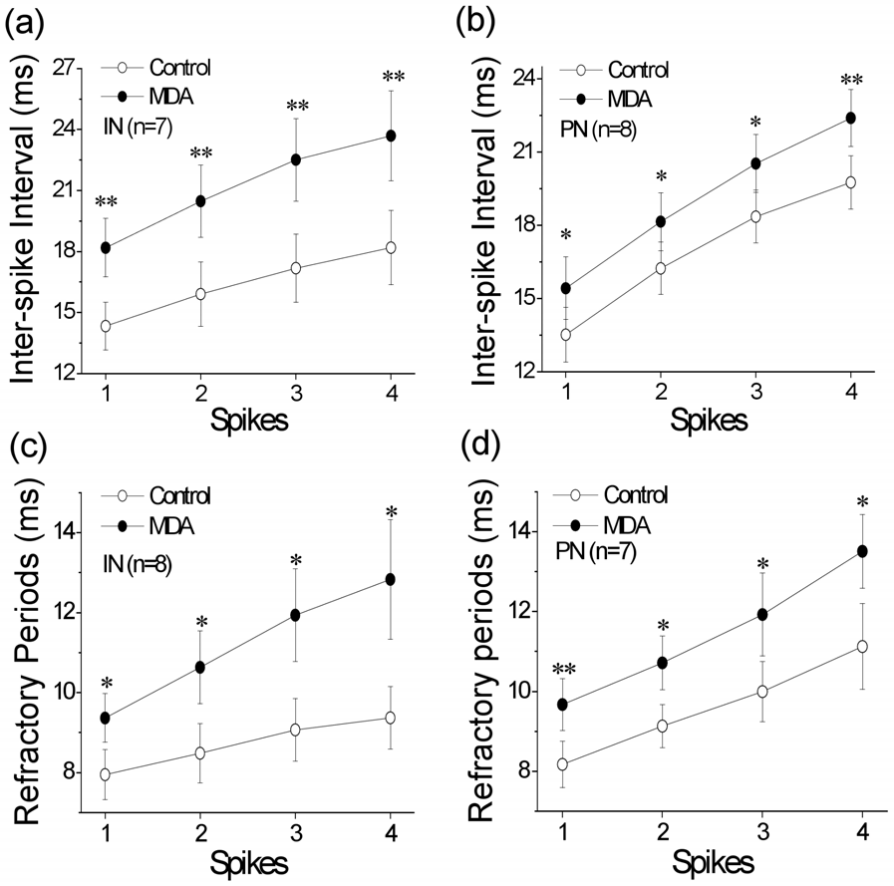
The comparison of inter-spike intervals (ISI) and the standard deviation of spike timing (SDST). ISI and SDST of pyramidal neurons (PN) and interneurons (IN) before and after 20 µM MDA treatments as indicated (* p<0.05, **p<0.01).

**Figure 4:**
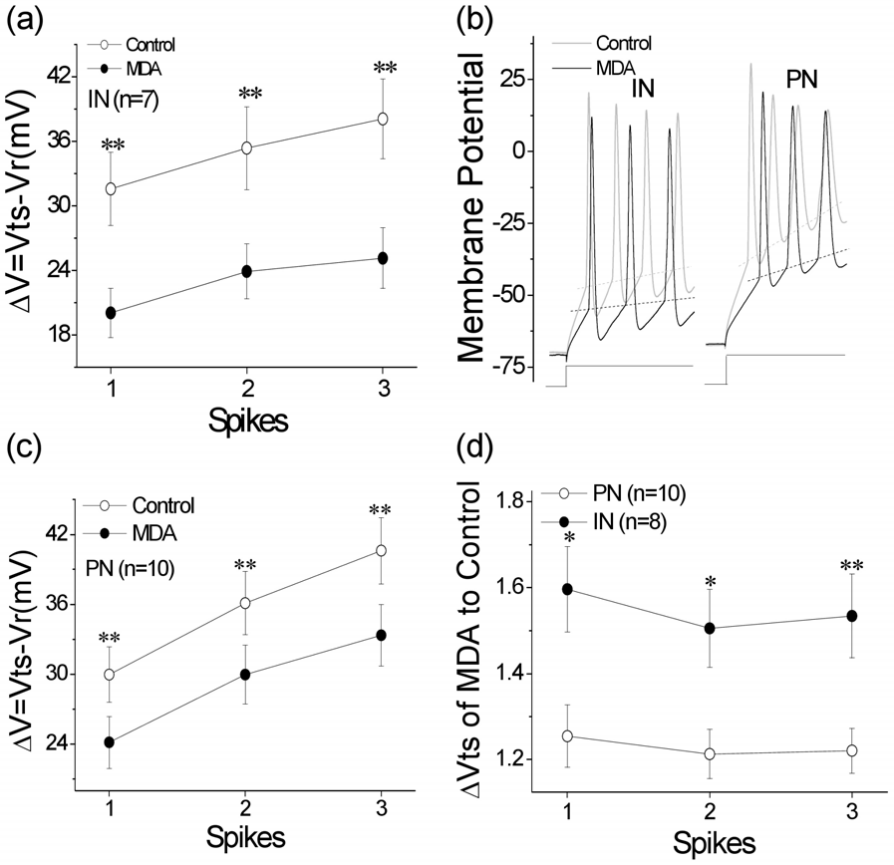
The comparison of threshold potentials of sequential spikes (ΔVts) and the absolute refractory periods (ARP). The threshold potentials (Vts) of IN and PN before and after 20 µM MDA treatment were shown in panel a and c. The membrance potentials were as indicated in panel b. ΔVts were presented in panel d (* p<0.05, **p<0.01).

To understand cellular mechanisms underlying MDA-induced sleep-like EEG and fatigue-like behaviors, we examined the influences of MDA on spike encodings in cortical neurons. Whole-cell patch clamp was conducted at pyramidal neurons and fast-spiking interneurons in cortical slices of turtles, and MDA (20 µM) was washed onto the brain slices by adding it into perfuse solution. MDA increased ISI of sequential spikes in pyramidal neurons and interneurons, especially in interneurons (Fig. 3; Table S1). As inter-spike intervals are mainly controlled by refractory periods [33], we tested if MDA prolonged ARP. The measurements of ARP were done by giving multiple pulses (Methods for details; Table S2). These results indicate that MDA attenuates the capacity of encoding sequential spikes through the prolongation of absolute refractory periods (Fig. 4).

### MDA breaks homeostasis between excitation and inhibition in brain networks

In terms of the mechanisms underlying the brain dysfunctions by MDA-mediated carbonyl stress, we examined whether MDA results in imbalance between glutamate and GABA activities in the brain of red-eared turtles. As glutamate and GABA are released from cortical pyramidal neurons and interneurons, respectively, an essential solution to this hypothesis includes the measurements in the levels of glutamate and GABA, as well as the sensitivities of pyramidal neurons and interneurons to the inputs while giving MDA.

The sensitivities of these neurons to excitatory inputs were measured based on the threshold potentials of sequential spikes. Figure 4 shows that MDA (20 µM) reduced threshold potentials (Vts) in pyramidal neurons and interneurons, but the change was more prominent in interneurons than in pyramidal neurons (Table S3). We also compared the changes in cellular sensitivity of interneurons vs. pyramidal neurons, in which the changes in threshold potentials (ΔVts) were calculated, i.e., threshold potentials under the condition of MDA treatments were divided by those under controls. Figure 4d shows ΔVts before and after MDA treatment at interneurons and pyramidal neurons indicating that an increase in the sensitivity of interneurons is more dominant than that of pyramidal neurons by MDA treatment (Table S4). This result well explains the role of MDA in inducing more GABA in brain tissues as follows.

### Changes in neurotransmitters during MDA treatment

To investigate the relation between the neurotransmitters and EEG suppression, changes in the Glu and GABA levels in the CNS during MDA treatment were detected using HPLC. The two time points (5 minutes and 35 minutes), at which the EEG showed significant changes, were chosen.

**Figure 5:**
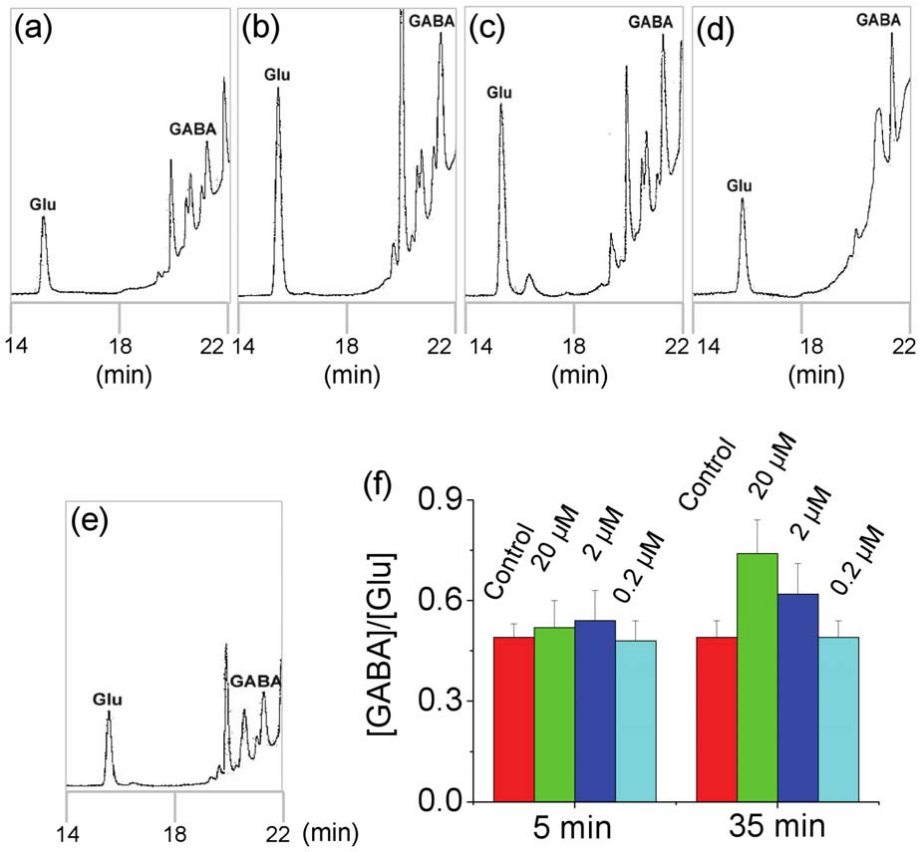
HPLC analysis of Glu and GABA content in turtles' brain under MDA treatment. Chromatograms were recorded in 5 minutes (panel a) and 35 minutes (panel b), under 20 µM MDA treatment. Then, chromatograms were recorded in 35 minutes under the treatment of 2 µM MDA (panel c); 0.2 µM MDA (panel d); and the phosphate buffer as control (panel e). The HPLC peak area was estimated to calculate the ratio of [GABA]/[Glu] from the brain extract of turtles at different time intervals under the MDA and control treatments (panel f).

Glu and GABA in a standard mixture of amino acids were clearly separated within 24 minutes. The retention time for Glu and GABA was about 15.8 minutes and 21.3 minutes, respectively (Fig. 5). The HPLC peak areas of GABA and Glu in the extract of turtle brain did not show significant changes in 5 minutes under 20 µM MDA treatment (Fig. 5a) compared to control group (Fig. 5e). In 35 minutes under the MDA treatment, corresponding to the strongest moment of slow wave EEG, the peak areas of both GABA and Glu showed statistical increment (p<0.01) (Fig. 5b, Tab. 3). The peak area of GABA increased much faster than that of Glu, thus the ratio between GABA and Glu increased significantly (p<0.01) (Fig. 5f). The turtles treated with 2 µM MDA showed a similar change but weaker than the 20 µM MDA treatment (Fig. 5c). However, the group treated with 0.2 µM MDA showed no statistical changes (Fig. 5d) compared to the control group (Tab. 3).

## Discussion

Turtles, as mentioned above, showed fatigue-like behavior after MDA administration. To explain primarily this characteristic behavior, we carried out (1) EEG of the red-eared turtles during the MDA treatment; (2) whole-cell recordings of brain slices; (3) examination of the actual MDA concentration in the turtle brain, and (4) analysis of the neurotransmitters by HPLC. Integrating with these experimental results, we attempted to figure out the relationship between the MDA and the CNS suppression under our experimental conditions.

As described previously [35], [36], [37], the EEG patterns exhibited by the chelonian reptiles are different from those in mammals during their sleep-wakefulness cycle [38]. But some studies show that the dorsal part of turtle cortex reveal obvious similarity in their connections and function to special areas in mammalian isocortex [39], [40], and the types of neurons found in turtle dorsal cortex (their morphology and neurotransmitter content) also show great similarity to those observed in mammals [40]. Furthermore, the chelonian reptile show two phases of sleep (quiet sleep phase and active sleep phase), which are respectively similar to slow wave sleep and rapid eye movement sleep in birds and mammals [35].

Large amplitude, arrhythmic spikes were reported to be a common attribute of the EEG of reptiles and some amphibians [41]. Generally, they were described in the association with behavioral quiescence (neck and limbs relaxed, eyes closed) or sleep [41]. In our study, after 2 µM or 20 µM MDA treatment, the EEG pattern shows the conversion from high frequency-low amplitude to low frequency-high amplitude. The EEG pattern of low frequency-high amplitude is similar to the sleep phase EEG of the reptile [35], [41]. After 20 µM MDA treatment, the EEG of the turtles further shows an almost complete suppression of EEG signal and the appearance of arrhythmic spikes. EEG analysis reveals statistical changes of power density in all 4 frequency bands (Figs. 1, 2 and Tab. 1). From 5 minutes to 35 minutes after MDA treatment, the power density of slow waves (theta wave and delta wave) increase faster than fast waves (beta wave and alpha wave). Heart rate of the turtles is significantly lower than that before treatment. As to the behaviors, after 20 µM MDA treatment, the turtles showed behavioral quiescence, with their neck and legs relaxed and extended from the shell, their heads rested on the experimental platform and their eyes closed. Reaction threshold to stimuli became significantly high. The changes of both the EEG and the behavior associated with behavioral quiescence or sleep and imply that the turtle CNS is suppressed [42].

We attempt to simulate the accumulated state of MDA in the turtle's brain, so the MDA concentration used in our study is higher than the normal concentration in the turtle's body. MDA was injected intrathecally into the spinal cavity of the turtle and entered the CNS via the cerebral-spinal circle. Following continuous accumulation of MDA, the MDA concentration increased continuously in the brain. The MDA end-concentration in the turtle's brain is examined. The MDA end-concentrations of 20 µM group (35 minutes), and 2 µM group (35 minutes) are respectively higher than that of time-matched control group (p<0.001), and are respectively 2.40 folds, and 1.89 folds of the effectual MDA concentration (20 µM) in whole-cell recordings.

The level of GABA increased relatively faster than that of Glu, and the [GABA]/[Glu] increased. About 5 minutes after the treatment, the MDA concentration in the brain reached 20 µM or more, the cerebral neurons (both the pyramidal neurons and interneurons) become more excitive. Further excitation of the cerebral neurons promoted the release of GABA and Glu. From 5 minutes to 35 minutes after 2 µM or 20 µM MDA treatment, GABA and Glu contents increased continuously. That is to say, the balance between the excitatory and inhibitory neurotransmitters was changed. In this period, the turtles' CNS was under a relative suppression state since the suppressed EEG was recorded. Following the metabolism of MDA in the turtle's brain, the MDA concentration decreased gradually, and the excitability of the cerebral neurons also decreased. So the levels of GABA and Glu also decreased and the balance between the excitatory and inhibitory neurotransmitters got back gradually. The turtles' CNS came back from the relative suppression state. The EEG also gradually came back to the basal condition from 35 minutes to 60 minutes after MDA treatment (not shown).

Glu and GABA is respectively the most important one of excitatory and inhibitory amino-acid neurotransmitters in the mammalian brain, and both of them play key roles in the sleep-wakefulness cycle [43], [44]. They are involved in accomplishment of critical functions in the reptile's brain [45]. The concentration ratio of [GABA]/[Glu] or vice versa [Glu]/[GABA] is usually used as an objective index to evaluate the CNS state. The MDA accumulation in the turtle's brain may change the balance among the neurotransmitters, so as to cause protective responses of the brain. As shown by HPLC analysis, in the period from 5 to 35 minutes after 20 or 2 µM MDA treatment, [GABA]/[Glu] increased statistically. It appears that the activity of the turtle brain is relatively suppressed because GABA increases faster than Glu. A number of studies reported that after sleep deprivation or excessive exercise, in which free radicals and unsaturated carbonyls appear largely, not only Glu and GABA but also other neurotransmitters may be protectively increased [43], [46], [47], [48]. Wang and coworkers [49] have reported that both the GABA contents and the ratio [GABA]/[Glu] show an increase in all the 3 examined brain regions (the frontal cortex, hypothalamus and brain stem) of the rats in 72 h sleep deprivation group (p<0.01) than those of control group. The Glu contents are also increased under their experimental conditions (p<0.05). Furthermore, as described [50], the contents of GABA in the cerebral tissue increase 15% under sleep state than that under consciousness state.

In terms of the mechanisms underlying the dysfunctions of cerebral neurons in brain networks by MDA from carbonyl stress, we tested our hypothesis that MDA initiates imbalances between GABA and glutamate activities, which break down the network neurons. In HPLC assay, we found that the levels of GABA in brain tissue treated by MDA were higher than those of glutamate. As these two neurotransmitters are released from interneurons and pyramidal neurons, the predominant increase in GABA level during MDA treatment may be caused by more excited interneurons. In examining the excitability in these two types of cerebral neurons, we observed that the attenuation of threshold potentials is more dominant in interneurons than pyramidal neurons. The more sensitivity of interneurons grants them releasing more GABA. Therefore, we propose that MDA elevation during carbonyl stress may suppress brain functions through breaking homeostasis between GABAergic and glutamatergic activities.

Mechanisms underlying MDA-induced sleep-like EEG and fatigue-like behaviors are likely due to neurons' dysfunction in cerebral cortex, which have been examined by patch clamp technique. In whole-cell recording, we found that MDA prolongs the refractory periods of sequential spikes mediated by voltage-gated sodium channels, and in turn inter-spike intervals, i.e., decrease a capacity of encoding action potentials, at cerebral interneurons and pyramidal neurons. The dysfunction of pyramidal cells causes fatigue-like behaviors, and the dysfunction of interneurons further weakens the encoding ability of pyramidal neurons (**) and sleep-like EEG.

Several studies demonstrated that neurotoxic 4-hydroxy nonenal inhibited various enzymes critical for the survival of neurons such as Na^+^-K^+^ ATPase [51], [52] and glutamate transporters [53]. The suppression of Na^+^-K^+^ ATPase could induce the slowing EEG, since the enzyme is the rate-limiting enzyme in determining EEG frequency [54]. So we speculate that the suppression of EEG signal also correlates with the interaction of MDA with critical proteins on the neuronal membrane. In addition, if reagents which block MA action could be shown to neutralize this response that would also significantly strengthen this work. To find some proper blockers and clarify the mechanism of these phenomena, further studies need to be done.

In summary, we found an attractive phenomenon that MDA triggered remarkable cerebral suppression of red-eared turtle and caused sleep-like EEG, and demonstrated that the turtle's brain has a stronger ability of anti-carbonyl stress than the mammal. According to the results, we further propose that similar phenomena should also appear when carbonyls are accumulated in mammalian brain, including the human being's.

## Supporting Information

Table S1 The values of Inter-spike Interval (ISI) for spikes 1∼4 in interneurons and pyramidal neurons (ms). * For Figure 3a, ISI values for corresponding spikes were statistically different before and after MDA treatment in interneurons (p<0.01). ** For Figure 3b, ISI values for corresponding spikes were statistically different before and after MDA treatment in pyramidal neurons (p<0.05).
(DOC)

Table S2 The absolute refractory period (ARP) values for spikes 1∼4 in interneurons and pyramidal neurons (ms). * For Figure 3c, ARP values for corresponding spikes were statistically different before and after MDA treatment in interneurons (p<0.05). ** For Figure 3d, ARP values for corresponding spikes were statistically different before and after MDA treatment in pyramidal neurons (p<0.05).
(DOC)

Table S3 The threshold potentials (Vts) values for spikes 1∼3 in interneurons and pyramidal neurons (mV). * For Figure 4a, Vts values for corresponding spikes were statistically different before and after MDA application in interneurons (p<0.01).** For Figure 4c, Vts values for corresponding spikes were statistically different before and after MDA application in pyramidal neurons (p<0.01).
(DOC)

Table S4 The comparison of threshold potentials of sequential spikes (ΔVts) values for spikes 1∼3 in interneurons and pyramidal neurons. * For Figure 4d, ΔVts values for corresponding spikes were statistically different at the interneurons and pyramidal neurons (p<0.01).
(DOC)
