# Supplementary material for: Malondialdehyde Suppresses Cerebral Function by Breaking Homeostasis between Excitation and Inhibition in Turtle Trachemys scripta
Source: PLoS One. 2010 Dec 22;5(12):e15325. doi: 10.1371/journal.pone.0015325 (PMC3008675; doi:10.1371/journal.pone.0015325)
Supplement: Table S1 — The values of Inter-spike Interval (ISI) for spikes 1∼4 in interneurons and pyramidal neurons (ms). * For Figure 3a, ISI values for corresponding spikes were statistically different before and after MDA treatment in interneurons (p<0.01). ** For Figure 3b, ISI values for corresponding spikes were statistically different before and after MDA treatment in pyramidal neurons (p<0.05). (DOC) [file pone.0015325.s001.doc]

Table S1. The values of Inter-spike Interval (ISI) for spikes 1~4 in interneurons and pyramidal neurons (ms).

|  |  | Spike 1 | Spike 2 | Spike 3 | Spike 4 |
| --- | --- | --- | --- | --- | --- |
| IN*  (n=7) | Control | 14.32±1.18 | 15.9±1.58 | 17.18±1.69 | 18.19±1.85 |
|  | MDA | 18.18±1.4 | 20.47±1.77 | 22.51±2.03 | 23.69±2.22 |
| PN**  (n=8) | Control | 13.51±1.12 | 16.23±1.07 | 18.35±1.08 | 19.75±1.10 |
|  | MDA | 15.42±1.29 | 18.15±1.19 | 20.54±1.18 | 22.39±1.16 |
