# Supplementary material for: Malondialdehyde Suppresses Cerebral Function by Breaking Homeostasis between Excitation and Inhibition in Turtle Trachemys scripta
Source: PLoS One. 2010 Dec 22;5(12):e15325. doi: 10.1371/journal.pone.0015325 (PMC3008675; doi:10.1371/journal.pone.0015325)
Supplement: Table S2 — The absolute refractory period (ARP) values for spikes 1∼4 in interneurons and pyramidal neurons (ms). * For Figure 3c, ARP values for corresponding spikes were statistically different before and after MDA treatment in interneurons (p<0.05). ** For Figure 3d, ARP values for corresponding spikes were statistically different before and after MDA treatment in pyramidal neurons (p<0.05). (DOC) [file pone.0015325.s002.doc]

Table S2. The absolute refractory period (ARP) values for spikes 1~4 in interneurons and pyramidal neurons (ms).

|  |  | Spike 1 | Spike 2 | Spike 3 | Spike 4 |
| --- | --- | --- | --- | --- | --- |
| IN*  (n=8) | Control | 7.94±0.63 | 8.48±0.74 | 9.06±0.78 | 9.37±0.78 |
|  | MDA | 9.36±0.61 | 10.63±0.91 | 11.93±1.16 | 12.82±1.5 |
| PN**  (n=7) | Control | 8.17±0.58 | 9.13±0.54 | 9.99±0.75 | 11.12±1.08 |
|  | MDA | 9.67±0.65 | 10.71±0.67 | 11.92±1.04 | 13.5±0.93 |
