# Supplementary material for: Malondialdehyde Suppresses Cerebral Function by Breaking Homeostasis between Excitation and Inhibition in Turtle Trachemys scripta
Source: PLoS One. 2010 Dec 22;5(12):e15325. doi: 10.1371/journal.pone.0015325 (PMC3008675; doi:10.1371/journal.pone.0015325)
Supplement: Table S3 — The threshold potentials (Vts) values for spikes 1∼3 in interneurons and pyramidal neurons (mV). * For Figure 4a, Vts values for corresponding spikes were statistically different before and after MDA application in interneurons (p<0.01).** For Figure 4c, Vts values for corresponding spikes were statistically different before and after MDA application in pyramidal neurons (p<0.01). (DOC) [file pone.0015325.s003.doc]

Table S3. The threshold potentials (Vts) values for spikes 1~3 in interneurons and pyramidal neurons (mV).

|  |  | Spike 1 | Spike 2 | Spike 3 |
| --- | --- | --- | --- | --- |
| IN*  (n=7) | Control | 31.57±3.4 | 35.36±3.85 | 38.07±3.68 |
|  | MDA | 20.1±2.29 | 23.9±2.55 | 25.14±2.83 |
| PN**  (n=10) | Control | 29.96±2.37 | 36.1±2.7 | 40.6±2.84 |
|  | MDA | 24.14±2.2 | 29.96±2.53 | 33.34±2.65 |
