# Supplementary material for: Malondialdehyde Suppresses Cerebral Function by Breaking Homeostasis between Excitation and Inhibition in Turtle Trachemys scripta
Source: PLoS One. 2010 Dec 22;5(12):e15325. doi: 10.1371/journal.pone.0015325 (PMC3008675; doi:10.1371/journal.pone.0015325)
Supplement: Table S4 — The comparison of threshold potentials of sequential spikes (ΔVts) values for spikes 1∼3 in interneurons and pyramidal neurons. * For Figure 4d, ΔVts values for corresponding spikes were statistically different at the interneurons and pyramidal neurons (p<0.01). (DOC) [file pone.0015325.s004.doc]

Table S4. The comparison of threshold potentials of sequential spikes (ΔVts) values for spikes 1~3 in interneurons and pyramidal neurons.

|  | Spike 1 | Spike 2 | Spike 3 |
| --- | --- | --- | --- |
| IN(n=7) | 1.60±0.1 | 1.51±0.09 | 1.53±0.1 |
| PN (n=10) | 1.25±0.07 | 1.21±0.06 | 1.22±0.05 |
